# Inhibition of RANKL-Induced Osteoclastogenesis by Novel Mutant RANKL

**DOI:** 10.3390/ijms22010434

**Published:** 2021-01-04

**Authors:** Yuria Jang, Hong Moon Sohn, Young Jong Ko, Hoon Hyun, Wonbong Lim

**Affiliations:** 1 Laboratory of Orthopaedic Research, Chosun University Hospital, Dong-Gu, Gwangju 61452, Korea; youria0526@nate.com (Y.J.); hmsohn@chosun.ac.kr (H.M.S.); yeasts@hanmail.net (Y.J.K.); 2 Department of Orthopaedic Surgery, Chosun University Hospital, Dong-Gu, Gwangju 61452, Korea; 3 Department of Biomedical Sciences Chonnam National University Medical School, Gwangju 61469, Korea; hhyun@chonnam.ac.kr; 4 Department of Premedical Science, College of Medicine, Chosun University, Dong-Gu, Gwangju 61452, Korea

**Keywords:** leucine-rich repeat-containing G-protein-coupled receptor 4, receptor activator of nuclear factor kappa-Β ligand, osteoclast, osteoporosis

## Abstract

Background: Recently, it was reported that leucine-rich repeat-containing G-protein-coupled receptor 4 (LGR4, also called GPR48) is another receptor for RANKL and was shown to compete with RANK to bind RANKL and suppress canonical RANK signaling during osteoclast differentiation. The critical role of the protein triad RANK–RANKL in osteoclastogenesis has made their binding an important target for the development of drugs against osteoporosis. In this study, point-mutations were introduced in the RANKL protein based on the crystal structure of the RANKL complex and its counterpart receptor RANK, and we investigated whether LGR4 signaling in the absence of the RANK signal could lead to the inhibition of osteoclastogenesis.; Methods: The effects of point-mutated RANKL (mRANKL-MT) on osteoclastogenesis were assessed by tartrate-resistant acid phosphatase (TRAP), resorption pit formation, quantitative real-time polymerase chain reaction (qPCR), western blot, NFATc1 nuclear translocation, micro-CT and histomorphological assay in wild type RANKL (mRANKL-WT)-induced in vitro and in vivo experimental mice model. Results: As a proof of concept, treatment with the mutant RANKL led to the stimulation of GSK-3β phosphorylation, as well as the inhibition of NFATc1 translocation, mRNA expression of TRAP and OSCAR, TRAP activity, and bone resorption, in RANKL-induced mouse models; and Conclusions: The results of our study demonstrate that the mutant RANKL can be used as a therapeutic agent for osteoporosis by inhibiting RANKL-induced osteoclastogenesis via comparative inhibition of RANKL. Moreover, the mutant RANKL was found to lack the toxic side effects of most osteoporosis treatments.

## 1. Introduction

Bone homeostasis is maintained due to a balance between bone resorption by osteoclasts and bone formation by osteoblasts [1,2]. However, excessive osteoclast activity leads to an imbalance between bone resorption and formation, which is often observed in patients with osteoporosis [3,4]. Receptor activator of nuclear factor kappa-Β ligand (RANKL) is a cytokine which is essential during osteoclast differentiation by regulating the differentiation and function of osteoclasts [5]. The drugs most commonly used to treat osteoporosis, such as alendronate (Binosto, Fosamax), risedronate (Actonel, Atelvia), ibandronate (Boniva), and zoledronic (Reclast, Zometa), are bisphosphonates [6,7,8]. These drugs are commonly used to decrease bone resorption by inhibiting the activity of osteoclasts [9,10]. However, long-term administration is known to cause side effects, such as jawbone necrosis and femoral fractures [11]. To overcome these types of side effects, the OPG-Fc fusion protein, which is capable of inhibiting RANKL activity, and an anti-RANKL antibody, were developed. The anti-RANKL antibody with superior pharmacokinetic properties was introduced as denosumab and approved as a therapeutic agent for osteoporosis and is currently used clinically. Denosumab is a fully human monoclonal antibody against RANKL and functions by blocking its binding to RANK, thereby inhibiting the development and activity of osteoclasts, followed by suppression of bone resorption. However, its side effects include hypocalcemia, infection, and skin reactions. In addition, as the drug inhibits bone turnover, it may also lead to jaw necrosis.

Recently, Leucine-rich repeat-containing G-protein-coupled receptor 4 (LGR4, also called GPR48) was shown to be another receptor of RANKL [12,13]. Studies have shown that LGR4 competes with RANK to bind RANKL and suppresses canonical RANK signaling during osteoclast differentiation [14]. RANKL binding to LGR4 activates the Gαq and GSK3-β signaling pathway, thereby suppressing the expression and activity of nuclear factor of activated T cells, cytoplasmic, calcineurin-dependent 1 (NFATC1) during osteoclastogenesis [14,15,16,17]. In addition, RANKL-RANK-NFATc1 signaling during osteoclast differentiation can directly induce the expression of LGR4, which competes with RANK for RANKL binding in osteoclast lineage cells [18,19]. LGR4 belongs to the LGR family, of which another two members, namely the thyroid-stimulating hormone receptor (TSHR) and follicle-stimulating hormone receptor (FSHR), regulate osteoclast differentiation and resorption [14].

Therefore, we generated a specific peptide modified from RANKL which could target LGR4 binding and RANK unbinding and investigated whether the novel peptide bound to LGR4 but not RANK and if it could inhibit osteoclastogenesis by inhibiting RANKL-RANK signaling. In a previous study, treatment with mutant-type mouse RANKL (mRANKL-MT) in in vitro and in vivo osteoporosis experimental models significantly inhibited the differentiation and production of osteoclasts, which suggested a potential crosstalk between RANKL–RANK and LGR4 signaling [20]. Therefore, in this study, we demonstrated that the novel peptide constructed binds to LGR4, which is another receptor for RANKL, and that it, via this ligand, acts to negatively regulate osteoclast differentiation and bone remodeling. These findings suggest that targeting LGR4 binding novel peptide as an agent for osteoporosis treatment by clarifying the effect and mechanism of action on osteoclast differentiation and bone resorption.

## 2. Results

### 2.1. Effect of Mutant RANKL on Osteoclast Differentiation In Vitro

**Figure 1:**
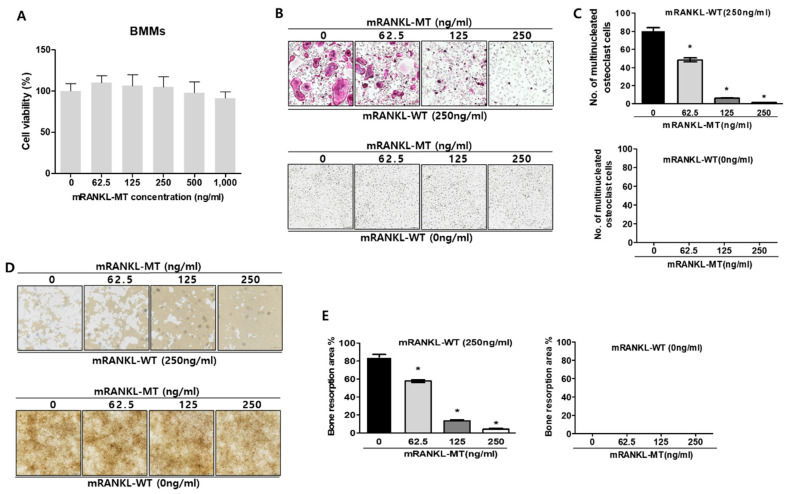

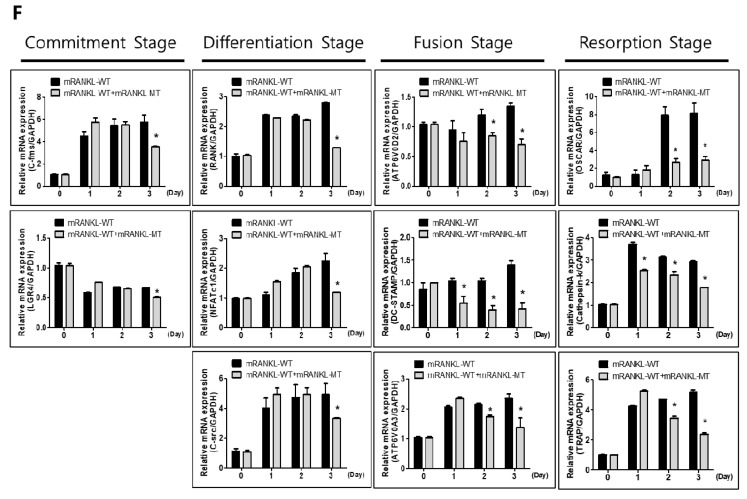
Effect of mutant RANKL (mRANKL-MT) on osteoclast differentiation in vitro (**A**). Cell viability was determined by 3-(4,5-dimethylthiazol-2-yl)-2,5-diphenyltetrazolium bromide (MTT) assay. BMMs cells were exposed to various doses (0, 62.5, 125, 250, 500 and 1000 ng/mL) of mutant RANKL. The results are presented as the mean ± SD of three independent experiments, and there shows no significant differences (*p* < 0.05) compared to the 0 ng/mL mutant RANKL-treated group. (**B**) A typical image of BMMs stained for TRAP (red) after treatment with various doses of mRANKL-MT (0, 62.5, 125, 250 ng) in the presence or absence of mRANKL-WT (250 ng/mL). (**C**). Numbers of multinucleated TRAP cells (BMMs) (red) (≥3 nuclei) in these cultures (n = 4); (**D**). BMMs were incubated in hydroxyapatite-coated plates with various doses of mRANKL-MT (0, 62.5, 125, 250 ng) in the presence or absence of mRANKL-WT (250 ng/mL). The cells attached to the plate were removed and imaged using a light microscope (**E**). The absorption area was quantified using the Image J software. * *p* < 0.01. (**F**) mRANKL-MT inhibits RANKL-induced osteoclast gene expression. BMMs were exposed to mRANKL-WT (2 µg/mL) or mRANKL-WT (2 µg/mL) + mRANKL-MT (2 µg/mL) for 3 days. Gene expression was determined by real-time PCR and normalized to the expression of GAPDH. The data come from three separate experiments and are expressed as the mean ± standard deviation (SD). * *p* < 0.01.

To determine whether mutant RANKL induces the changes of cell viability, MTT cell viability assay was carried out in dose dependent manners of mutant RANKL (Figure 1A). As a matter of result, no changes of cell viabilities were observed even in the 1000 ng/mL mutant RANKL treatment.

To evaluate the inhibitory effect of mRANKL-MT on osteoclastogenesis in vitro, BMMs were treated with various concentrations of mRANKL-MT in the presence or absence of mRANKL-WT (250 ng/mL). The number of BMMs which differentiated into mature, TRAP-positive, multinucleated osteoclasts significantly decreased in a dose-dependent manner in the mRANKL-MT-treated group in the presence of mRANKL-WT (250 ng/mL) (Figure 1B,C). However, no trap-positive cells were observed in the mRANKL-WT (0 ng/mL)-treated group. In addition, the inhibition of osteoclast activity by mRANKL-MT was confirmed through a bone resorption assay. Our results showed that the bone resorption pit was decreased in mRANKL-MT-treated BMMs in the presence of mRANKL-WT in a dose-dependent manner. Moreover, we detected the inhibition of osteoclast activity in BMMs treated with the same concentration of mRANKL-MT (Figure 1D,E).

To evaluate the effect of mRANKL-MT on the mRNA expressions of osteoclastogenesis-related genes, we investigated the expression of several osteoclast-specific genes in both mRANKL-WT and mRANKL-WT + mRANKL-MT-treated BMMs (Figure 1F), Our results showed a significant decrease in c-fms, LGR4, RANK, NFATc1, and C-src mRNA expression which were known to associate with commitment and differentiation of osteoclast in mRANKL-WT + mRANKL-MT-treated BMMs on day 3 post-treatment. Moreover, ATP6V0d2, DC-STAMP, OSCAR, Cathepsin K, TRAP, and ATP6V0A3s mRNA expression which were known to related with fusion and activity of osteoclast was also found to be decreased on day 1 or 2 post-treatment. Overall, these results demonstrate that RANKL-induced mRNA expression of osteoclastogenesis-related genes was inhibited by mRANKL-MT.

### 2.2. Effect of Mutant RANKL on the RANK-RANKL/LGR4-LANKL Signaling Cascade

**Figure 2:**
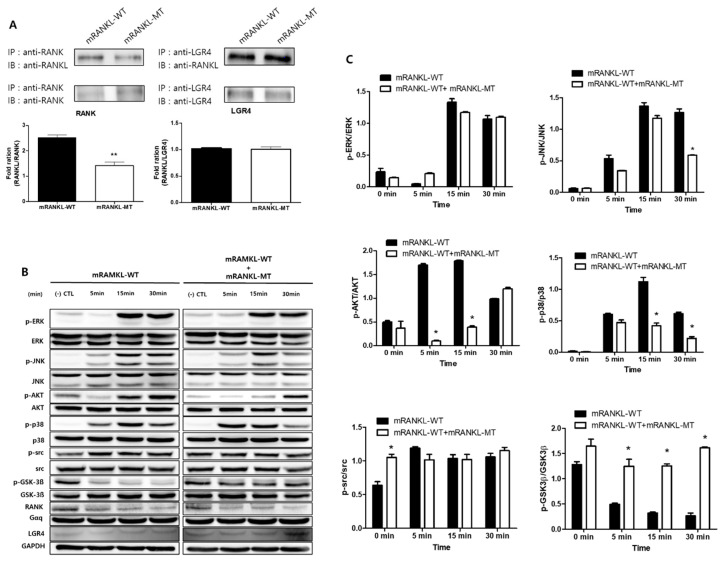
Comparative inhibition of osteoclastogenesis by RANKL variant. (**A**) Co-immunoprecipitation for RANK or LGR4 binding RANKL variant in BMMs. Each blot was obtained under the same experimental conditions. The densitometric analysis is represented as the mean ratio ± standard deviation (SD) of three separate experiments. * *p* < 0.01, control group vs. mRANKL-WT group; ** *p* < 0.01 mRANKL-WT group vs. mRANKL-WT + mRANKL-MT group. (**B**) Western blot analysis of the RANK and LGR4 signaling cascades. GAPDH was used as a loading control. (**C**) A densitometric analysis of protein phosphorylation in ERK, JNK, AKT, P38, src and GSK-3β. Results are representative of three separate experiments with comparable results. BMMs were exposed to mRANKL-WT (2 µg/mL) or mRANKL-WT (2 µg/mL) + mRANKL-MT (2 µg/mL) for various time intervals.

To investigate the interaction of mRANKL variant and RANK, Co-IP interaction assay was carried out between wild/mutant type of RANKL and RANK/LGR4 (Figure 2A). It showed that the wild type RANKL (mRANKL-WT) binds strongly to RANK in compared with mutant RANKL (mRANKL-MT), apparently. However, both of mRANKL-WT and mRANKL-MT could bind strongly to LGR4.

To evaluate the effect of mutant RANKL on the RANK-RANKL/LGR4-RANKL signaling cascade, we investigated whether treatment with mRANKL-WT or mRANKL-WT + mRANKL-MT for different durations led to the RANK and LGR4 signaling cascade inducing MAPK, AKT, src, and GSK-3β phosphorylation (Figure 2B). Treatment with mRANKL-MT in the presence of mRANKL-WT was found to significantly inhibit AKT, P-38 and GSK-3β phosphorylation (Figure 2C).

These results proposed that mRANKL-MT strongly transfer the signal from LGR4 only in compared with signal from both of mRANKL-WT/RANK and mRANKL-WT/LGR4 and may play an inhibitory role against RANKL-RANK signaling cascade.

### 2.3. Effect of Mutant RANKL on NFATc1 Translocation to the Nucleus

**Figure 3:**
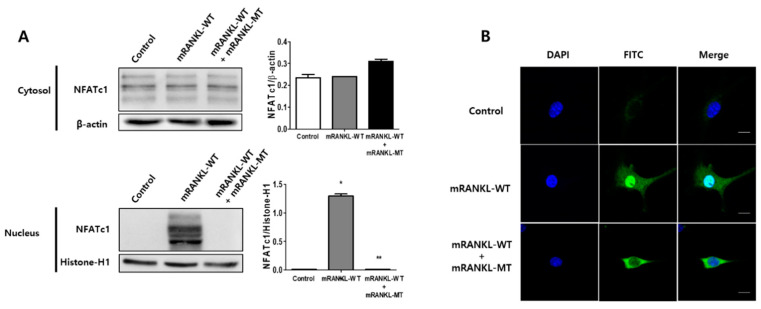
The inhibitory effect of mRANKL-MT on NFATc1 translocation in BMMs. (**A**) NFATc1 nuclear translocation was analyzed by western blot in the cytoplasmic and nuclear fractions. Histone-H1 and β-actin were used as loading controls for the nuclear and cytoplasmic fractions, respectively. The densitometric analysis of NFATc1 in the cytoplasmic and nuclear fractions is represented as the mean ratio ± standard deviation (SD) of three separate experiments. * *p* < 0.01, control group vs. mRANKL-WT group; ** *p* < 0.01 mRANKL-WT group vs. mRANKL-WT + mRANKL-MT group. (**B**) NFATc1 nuclear translocation under confocal microscopy. Immunofluorescence images were acquired by staining for NFATc1 (green) and the nucleus (blue). Magnifications are 200Χ. Size bar is 20 μm.

To evaluate the effect of mutant RANKL on NFATc1 translocation to the nucleus, we examined the effect of mRANKL-MT treatment on the nuclear localization of NFATc1. NFATc1 nuclear translocation was not detected in the mRANKL-WT group (Figure 3A). However, in the presence of mRANKL-WT, mRANKL-MT resulted in a significant inhibition of NFAc1 translocation. In the confocal microscopy analysis, NFATc1 was present in the nuclei of mRANKL-WT treated BMMs (Figure 3B) but was not detected in the nuclei and cytosol of BMMS treated with mRANKL-MT in the presence of mRANKL-WT.

These results further support our hypothesis that mRANKL-MT inhibits osteoclast formation, which is stimulated by mRANKL-WT and can be considered an effective competitive inhibitor for mRANKL-WT.

### 2.4. Effect of Mutant RANKL on RANKL-Induced Mice

**Figure 4:**
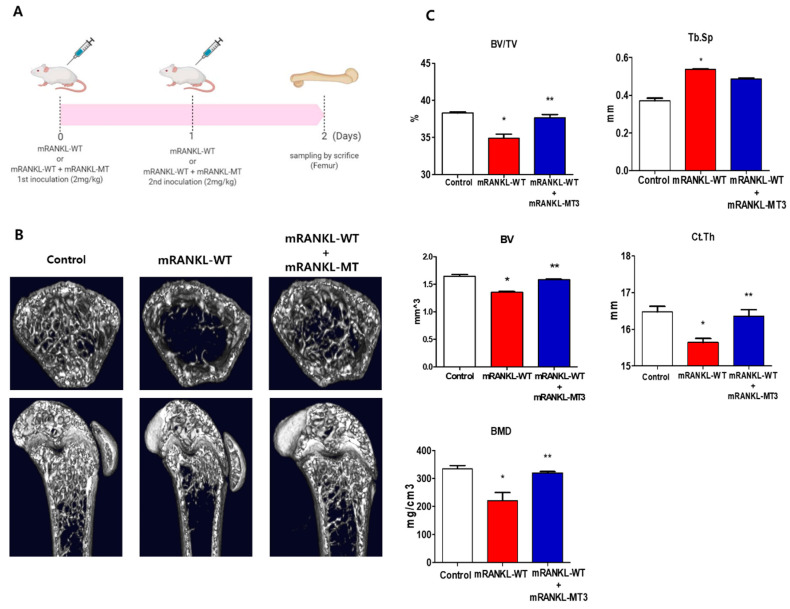
3D micro-computed tomography (micro-CT) images of mouse femurs (**A**). Time schedule for immunization and sampling in mRANKL-WT or mRANKL-WT+mRANKL-MT-treated mouse. (**B**) Representative X-ray and micro-CT images of the distal femurs of intact mice (control), RANKL-induced osteoporosis mice (mRANKL-WT), and mRANKL-WT-induced osteoporosis mice treated with mRANKL-MT (**C**). Bone volume/total volume (BV/TV), trabecular thickness (Tb.Th), trabecular spacing (Tb/sp), Bone Volume (BV), Cortical thickness (Ct/Th), Bone Mineral Density (BMD). * *p* < 0.01, control group vs. mRANKL-WT group; ** *p* < 0.01 mRANKL-WT group vs. mRANKL-WT + mRANKL-MT group.

To investigate the effect of mtRANKL on bone lysis, healthy mice were treated with mRANKL-MT in the presence of mRANKL-WT and their femur bones were then examined by micro-CT. Based on the previous study, the mRANKL-WT-treated mouse was used as an osteoporosis animal model to examine the therapeutic effect of mRANKL-MT protein on osteoporosis (Figure 4A). Mice in the mRANKL-WT group were found to exhibit significant bone loss, while mice in the mRANKL-WT + mRANKL-MT group exhibited little bone loss (Figure 4B). The BMD, BV/TV, Tb.Th, BV. Ct.Th, and other bone scores were assessed by quantitative micro-CT. As expected, the BMD, BV/TV, and Tb.Th were found to be decreased in mRANKL-WT mice and were significantly recovered by mRANKL-MT treatment. Thus, these results clearly demonstrate the therapeutic effects of mRANKL-MT against osteoporosis (Figure 4C).

### 2.5. Effect of mRANKL-WT + mRANKL-MT Decreased the Formation of Osteoclasts in Mice

**Figure 5:**
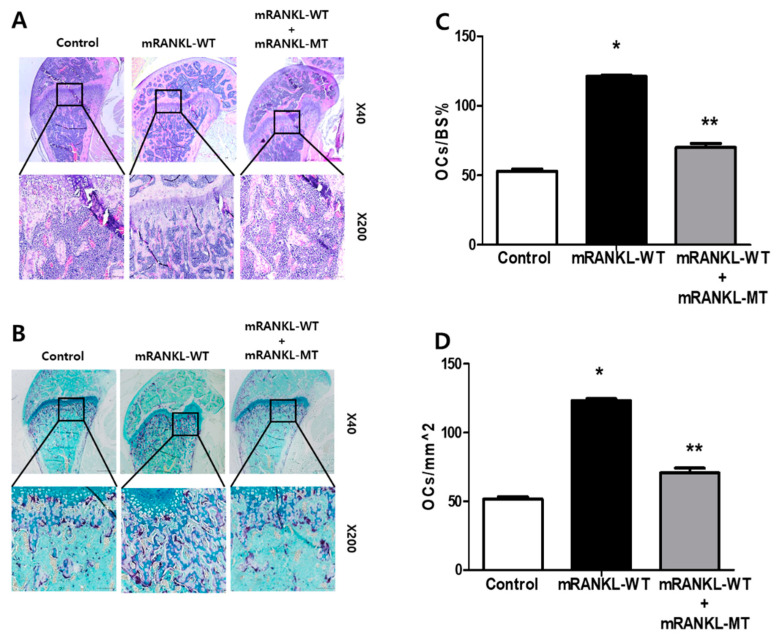
mRANKL-MT decreases the formation of osteoclast in mice (**A**) Histomorphometric analysis images. Magnifications are 20×. Size bar is 500 μm. (**B**) TRAP staining images of the femurs. Magnifications are 100×. Size bar is 50 μm. (**C**,**D**) Parameters of the femur osteoclasts. Oc.S/BS, osteoclast surface per bone surface; Oc.N/BS, osteoclast number per bone surface. Values were expressed as means ± standard deviation (SD). * *p* < 0.01 control vs. mRANKL-WT, ** *p* < 0.01 mRANKL-WT vs. mRANKL-WT + mRANKL-MT.

The trabecular bone of mice in the mRANKL-WT group was histologically analyzed by H&E staining and found to be thin and with a network structure that was severely disrupted and fragmented (Figure 5A). However, the trabecular bone of mice from the mRANKL-WT + mRANKL-MT group exhibited a normal arrangement pattern, with a thick and dense network with minimal spaces, which was similar to that of the control group. In addition, we stained histological sections of TRAP staining experiments and found that the samples from the mRANKL-WT + mRANKL-MT group exhibited fewer TRAP-positive osteoclasts compared to the mRANKL-WT group (Figure 5B). To quantify the data, we calculated the ratio between the TRAP-positive area and the trabecular bone surface (OCs/BS%), as well as the ratio between the number of osteoclasts and the bone area (OCs/mm^2^), for each group. The results confirmed that both the OCs/BS% and OCs/mm^2^ values were significantly smaller in the mRANKL-WT+mRANKL-MT group than those of the mRANKL-WT group (Figure 5C,D).

## 3. Discussion

Osteoporosis is a disease characterized by low bone mass, deterioration of the bone tissue, and disruption of the bone microarchitecture [21]. Most conventional drugs used to treat osteoporosis, such as bisphosphate, SERM, calcitonin, and estrogen inhibit bone resorption, while calcium, vitamin D, and others are also frequently used as supplements [22]. In the past two decades, it has been well demonstrated that RANKL (also known as TNFSF11) binding to its receptor RANK (also known as TNFRSF11A) drives osteoclast development and it is considered a crucial target protein for osteoporosis treatments [23]. Considering the signaling cascade of RANKL-RANK and its role in osteoclast differentiation, the application of denosumab (human anti-RANKL mAb) in clinical therapies for osteoporosis and the validation of a novel peptide which targets RANKL is theoretically possible.

As such, the development of a competitive inhibitor of the RANKL-RANK signaling cascade may be an alternative therapeutic approach to treating osteoporosis. Recently, LGR4 (also called GPR48) was reported as another receptor for RANKL [24]. Moreover, studies have shown that this novel RANKL receptor competes with RANK to bind RANKL and suppresses canonical RANK signaling during osteoclast differentiation [14]. Thus, LGR4 signaling was found to inhibit RANKL-induced osteoclast differentiation by blocking RANK–TRAF6 signaling, as well as through Gαq- and GSK3-β mediated inhibition of NFATC1 [14].

**Figure 6:**
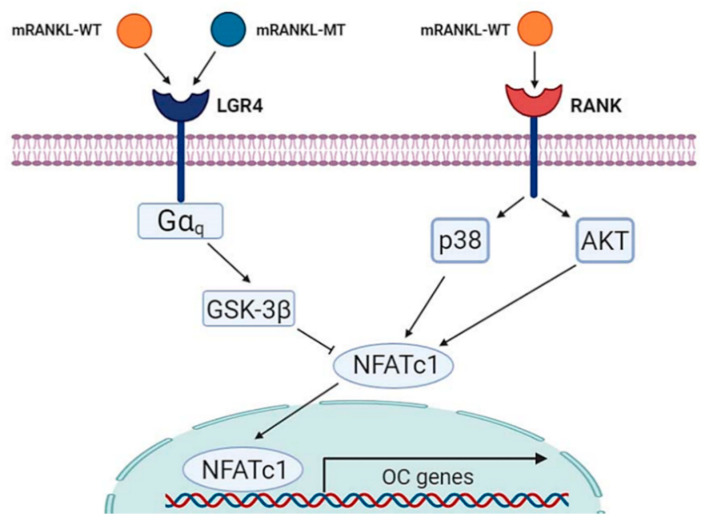
Schematic diagram of mRANKL-MT in inhibitory effect against mRANKL-WT/RANK signaling during osteoclastogenesis.

In the present study, based on this mechanism, the RANKL protein was mutated at the RANK binding site into similar amino acid residues to maintain the crystal structure of wild-type RANKL reported in a previous study and we investigated whether this mutant RANKL protein binds to LGR4 and acts as a competitive inhibitor of RANKL-RANK signaling to inhibit osteoclastogenesis. We found that that the wild type RANKL could bind both LGR4 and RANK but mutated RANKL binds only LGR4. As a proof of concept, treatment with mRANKL-MT inhibited osteoclast differentiation in the presence of wild-type RANKL both in vitro and in vivo, which suggests that this may be a competitive inhibitor of RANKL (Figure 6). RANKL is known to regulate osteoclast survival by reducing the expression of the death receptor Fas in mature osteoclasts [25]. However, mature osteoclasts ultimately undergo apoptosis in a RANKL-containing environment, which suggests the existence of a RANKL-induced signaling pathway that limits the survival of mature osteoclasts [26]. Our data suggest that LGR4 may be a pivotal player of the negative-feedback mechanism that controls the activities of osteoclast. The LGR4 signaling cascade was shown to activate the Gαq and GSK-3β signaling pathways, that act to inhibit the expression and activity of NFATC1 during osteoclast differentiation. Furthermore, the extracellular domain of LGR4 is known to have a lower binding affinity than RANKL, which indicates that LGR4-mRANKL-MT binding had little physiological side effects on osteoclast differentiation in normal mice. This suggests that the minimal side effects induced by the binding of LGR4-mRANKL-MT in normal mice could be due to endogenous RANKL competition [14]. Especially in the present study, LGR4 expression was found to be decreased during osteoclast differentiation and thus, targeting LGR4 may have an effect from pre-osteoclasts. Additionally, based on the significant reduction in the mRNA expression of genes involved in late-stage osteoclastogenesis, such as OSCAR and Cathepsin K, in mRANK-MT treated BMMs, mRANKL-MT could be an efficient inhibitor of wild-type RANKL as a specific target of active osteoclast.

In summary, the novel peptide mRANKL-MT induces a competitive inhibition effect against RANKL during osteoclastogenesis. Moreover, mRANKL-MT itself is involved in the LGR4-induced mediation of the RANKL–NFATC1 signaling cascade and negative-feedback mechanism to control osteoclast activities. The critical role of the protein triad RANK–RANKL in osteoclastogenesis has made their binding an important target for the development of drugs against osteoporosis. As a proof of concept, the mutant RANKL protein constructed in this study was shown to induce GSK-3β phosphorylation, as well as the inhibition of NFATc1 translocation, mRNA expression of TRAP and OSCAR, TRAP activities, and bone resorption, in RANKL-induced mouse models.

The results of this study demonstrate that the mutant RANKL protein has potential as a therapeutic agent for osteoporosis, by inhibiting RANKL-induced osteoclastogenesis via competitive inhibition of RANKL. Moreover, it was found to lack the toxic side effects which are common in osteoporosis treatments.

## 4. Materials and Methods

### 4.1. RANKL Variant Production by Site-Directed Mutagenesis

The polymerase chain reaction (PCR) product was cloned into the NdeI/XhoI site of the pGEX-4T-1 vector (Promega, Madison, WI, USA) and mutations at positions 180, 189–190, and 223–224 were introduced using the following megaprimers: mRANKL-NdeI, 5′-CATATGAAGCCTGAGGCCCAGCCATTTGC-3′; mRANKL-XhoI, 5′-CTCGAGGTCTATGTCCTGAACTTTGAAAGCC-3′; mRANKL(K180R)-F, 5′-CCCATCGGGTTCCCATCGAGTCACTCTGTCCTCTTG-3′; mRANKL(K180R)-R, 5′-CAAGAGGACAGAGTGACTCGATGGGAACCCGATGGG-3′; mRANKL(D189I, R190K)-F, 5′-CTCTTGGTACCACATCAAGGGCTGGGCCAAGAT-3′; mRANKL(D189I, R190K)-R, 5′-ATCTTGGCCCAGCCCTTGATGTGGTACCAAGAG-3′; mRANKL-MT3 (H223F, H224Y)-F, 5′-AACATTTGCTTTCGGTTTTATGAAACATCGGGAAGCG-3′, mRANKL-MT3 (H223F, H224Y)-R, 5′-CGCTTCCCGATGTTTCATAAAACCGAAAGCAAATGTT-3′, The PCR product was transformed into Escherichia coli BL21-Gold competent cells (Agilent, Santa Clara, CA, USA) by electroporation (5 ms, 12.5 kV/cm). The transformed E. coli cells were cultivated in Luria–Bertani (LB) broth supplemented with ampicillin (50 μg/mL, T&I, Daejeon, Korea). The plasmid was then purified using the QIAprep Spin Miniprep Kit (Qiagen, Valencia, CA, USA). The sequence of the cloned product was confirmed via a commercial sequencing service (SolGent Co., Daejeon, Korea). All sequence analyses were carried out using the Vector NTI Advance 9.1.0 software (Invitrogen, Carlsbad, CA, USA).

**Figure 7:**
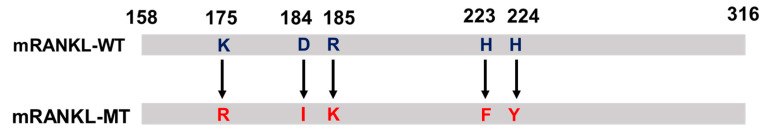
Protein sequence of mutant RANKL (mtRANKL), including the K180R, D189I, R190K, H223F, and H224Y transformants.

Alignment of the mRNA sequence of mRANKL-MT with mRANKL-WT (NCBI accession number AAB86812) revealed the region amplified with the selected primers. The mRANKL-WT and mRANKL-MT sequence encoded the full length 158 amino acid target region, which included residues 158 to 316 (Figure 7). To create a point mutation at positions 180 Lys, 189 Asp-190 Arg, and 223 His-224 His, the matched codon that probably corresponded to the similar structure of the side chain was transformed into 180 Arg, 189 Ile-190 Lys, and 223 Phe-224 Tyr, respectively.

### 4.2. mRANKL-MT Purification

The recombinant plasmid carrying mRANKL-WT and mRANKL-MT were expressed from a single E. coli BL21-Gold colony using previously described methods [20]. Briefly, a single colony carrying the recombinant plasmid was inoculated in 20 mL of LB medium supplemented with ampicillin (50 μg/mL) and incubated at 37 °C with agitation at 200 rpm for 15 h. Afterwards, 10 mL of the culture was inoculated in an Erlenmeyer flask containing 1 L of LB medium supplemented with 50 μg/mL of ampicillin. The cells were incubated at 37 °C and 180 rpm until the absorbance at 600 nm (OD600) reached a value of ≈ 1.0. Isopropyl β-D-1-thiogalactopyranoside (IPTG) was then added at a concentration of 0.1 mM to induce protein expression and the cells were further cultured for 6 h. After induction, cultures were centrifuged at 5600× *g* for 20 min at 4 °C and the cell pellets were stored at −20 °C until further use. To prepare a bacterial lysate for affinity column chromatography, the pellet was resuspended in a lysis buffer (phosphate buffered saline [PBS], 1 mM ethylenediaminetetraacetic acid [EDTA] pH 8.0, 0.1% Tween 20, 20 µM phenylmethylsulfonyl fluoride [PMSF]) and sonicated (Vibra Cell VCX500; Sonics & Materials, Inc., Newtown, CT, USA) on an ice bed. After sonication, the recombinant GST fusion RANKL protein was purified using a Glutathione Sepharose TM 4M resin column (GE Healthcare, Uppsala, Sweden) according to the manufacturer’s protocol. The eluted protein was dialyzed against a dialysis buffer (20% *v*/*v* glycerol in PBS) in a 10,000 MW Slide-A-Lyzer Dialysis cassette (Thermo Fisher Scientific, Waltham, MA, USA). The purified protein was then vacuum concentrated (Savant Instruments, Holbrook, NY, USA) and analyzed via sodium dodecyl sulfate-polyacrylamide gel electrophoresis (SDS-PAGE). Protein concentrations to calculate yields were determined using the Bradford assay. For endotoxin removal, a high-capacity endotoxin removal resin (Pierce Biotechnology Inc., Rockford, IL, USA) was used to remove contaminating lipopolysaccharides (LPS) from the recombinant protein. The endotoxin level in the isolated RANKL protein was 0.48 EU/mL resulting in 0.92 EU per 1 mg administered to each mouse. The recombinant protein samples were then resolved by electrophoresis. After separation, the gel was stained with Coomassie brilliant blue G-250. To determine the purity and recovery rate of the recombinant protein, a stained gel loaded with a fixed amount of protein was imaged using a digital scanner (EPSON, Chicago, IL, USA) at 300 dpi.

### 4.3. TRAP Assay

Bone marrow cells (BMCs) were flushed from the tibias and femurs of 6–8 weeks-old female C57BL/6 mice, cultured in Minimum Essential Medium Eagle—Alpha Modification (alpha-MEM) supplemented with 10% fetal bovine serum (FBS) and 30 ng/mL macrophage colony-stimulating factor (M-CSF) (R&D Systems, Minneapolis, MN, USA) after treatment with red blood cell (RBC) lysis buffer (Gibco, Gaithersburg, MD, USA). After being incubated for 3 days, non-adherent cells were removed, and the adherent cells were used as bone marrow macrophages (BMMs). Subsequently, osteoclast differentiation was induced by adding 30 ng/mL of M-CSF and various concentrations of RANKL (R&D systems) and further culturing the cells in the presence or absence of antibody or antiserum for 4 days. The multinucleated cells were then stained for tartrate-resistant acid phosphatase (TRAP) (KAMIYA BIOMEDICAL Co., Seattle, WA, USA) and cells with more than 3 TRAP-positive nuclei were identified as osteoclasts.

### 4.4. Bone Resorption Assay

To observe bone resorption in vitro, BMMs were cultured to Corning^®^ Osteo Assay Surface 96-well Multiple Well Plates (Sigma, Cat no. CLS3988) with M-CSF plus RANKL for 6 days with or without different concentrations of antibody. The plates were then washed with pure water.

### 4.5. Real-Time Reverse Transcription PCR (qRT-PCR) Analysis

The BMMs were incubated in the presence of 30 ng/mL of M-CSF and 75 ng/mL of sRANKL (R&D systems) or produced RANKL for indicated times in 6-well plates. Total RNA was extracted from the cells using the TRIzol^®^ reagent (Invitrogen; Thermo Fisher Scientific, Inc.). The cDNA was then obtained from 2 μg of total RNA using the ReverTra Ace^®^ qPCR RT Master Mix (TOYOBO). mRNA levels were measured via qRT-PCR and GAPDH was used as an endogenous control. The qRT-PCR was conducted on a CFX Connect Real-Time PCR Detection System (BIO-RAD, Hercules, CA, USA) using a 20 μL reaction mixture containing 10 μL of IQ SYBR Green Supermix (BIO-RAD), 10 pmol of the forward primer, 10 pmol of the reverse primer, and 1 μg of cDNA. The sequences of the primers used to target the various genes are listed as below: c-fms-F;5′-GCG ATG TGT GAGCAA TG CAG T-3′, c-fms-R;5′-GAG CCG TTT TGC GTA AGA CCT G-3′, LGR4-F;5′-TAGGATTCAC TGGGACCCTA GTGCT-3′, LGR4-R;5′-CAGTTTGTGA AGATGAGCCA AGA-3′, RANK-F;5′-CCA GGG GAC AAC GGA ATC A-3′, RANK-R;5′-GGC CGG TCC GTG TAC TCA TC-3′, NFATc1-F;5′-CAA CGC CCT GAC CAC CGA TAG-3′, NFATc1-R;5′-GGC TGC CTT CCG TCT CAT AGT-3′, C-src-F; 5′-ATG GGC TCT CCT GTC AAC AC-3′, C-src-R; 5′- GGC TGC CAA AAT AAA CTC CA-3′, Atp6v0d2-F;5′-GAA GCT GTC AAC ATT GCA GA-3′, Atp6v0d2-R;5′-TCA CCG TGA TCC TTG CAG AAT-3′, DC-STAMP-F:5′-TGG AAG TTC ACT TGA AAC TAC GTG-3′, DC-STAMP-R;5′-CTC GGT TTC CCG TCA GCC TCT CTC-3′, ATP6v0a3-F;5′-CGC CAC AGA AGA AAC ACT CA-3′, ATP6v0a3-R;5′-CCC AGA GAC GCA AGT AGG AG-3′, OSCAR-F;5′-CTG CTG GTA ACG GAT CAG CTC CCC AGA-3′, OSCAR-R;5′-CCA AGG AGC CAG AAC CTT CGA AAC T-3′, Cathepsin K-F;5′-TGT ATA ACG CCA CGG CAA A-3′, Cathepsin K-R;5′-GGT TCA CAT TAT CAC GGT CAC A-3′, TRAP-F; 5′-TAC CGT TGT GGA CAT GAC C-3′, TRAP-R; 5′-CAG ATC CAT AGT GAA ACC GC-3′, and β-actin-F;5′-GTC CCT CAC CCT CCC AAA AG-3′, β-actin-R;5′-GCT GCC TCA ACA CCT CAA CCC-3′.

### 4.6. Co-Immunoprecipitation Assay

BMM cells were incubated with 500 ng/mL of mRANKL-WT or mRANKL-MT3 at 37 °C for 45 min, and then cells were lysed in lysis buffer (20 mM Tris-HCl pH 7.6–8.0, 100 mM sodium chloride NaCl, 300 mM sucrose, 3 mM MgCl_2_ [buffer A]; and 20 mM Tris pH 8.0, 100 mM NaCl, 2 mM ethylenediaminetetraacetic acid [buffer B]). Whole cell lysates obtained by centrifugation were incubated with antibodies specific for RANK (Cell Signaling Technology, Danvers, MA, USA, #14373S) and LGR4 (MyBioSource, San Diego, CA, USA, #MBS468030) (dilution 1:100) and protein A Sepharose beads (Amersham Biosciences, Little Chalfont, UK) for 2 h at room temperature. The immune complexes were washed three times using wash buffer and examined by western blotting.

### 4.7. Western Blot Analysis

Cells were serum-starved for 8 h and treated with 2 μg/mL of mRANKL-MT at 5, 15, and 30-min intervals. The cells were then lysed in 5X SDS-loading buffer supplemented with protease and phosphatase inhibitor cocktails. Approximately 30 mg of the cell lysate was separated via 10% SDS-PAGE and transferred onto a polyvinylidene difluoride (PVDF) membrane (Amersham, Piscataway, NJ, USA). Each membrane was blocked for 30 min with a blocking solution containing 5% skim milk in Tris-buffered saline containing Tween-20 (TBST, 2.42 g/L Tris-HCl, 8 g/L NaCl, 0.1% Tween-20, pH 7.6) and rinsed with TBST. The membrane was then incubated overnight at 4 °C with the appropriate primary antibodies, namely p-Akt (1:1000; 9271S; Cell Signaling Technology) Akt (1:1000; 9272S; Cell Signaling Technology), p-p38 (1:1000; 9211S; Cell Signaling Technology), p38 (1:1000; 9212S; Cell Signaling Technology), p-ERK (1:1000; 9101S; Cell Signaling Technology), ERK (1:1000;9102S; Cell Signaling Technology), p-JNK (1:1000; 9251S; Cell Signaling Technology), JNK (1:1000; 9252S; Cell Signaling Technology), p-GSK-3β (1:1000; 9336S; Cell Signaling Technology), GSK-3β (1:1000; 9315S; Cell Signaling Technology), p-Src (1:1000; 2105S; Cell Signaling Technology), Src (1:1000; 2108S; Cell Signaling Technology), RANK (1:1000; 4845S; Cell Signaling Technology), Gαq (1:1000; 14373S; Cell Signaling Technology), and LGR4 (1:500; MBS468030; MyBioSource). A mouse monoclonal immunoglobulin G (IgG) antibody specific for GAPDH (1:1000; 2118S; Cell Signaling Technology) was used as a control. The membrane was then rinsed with TBST and protein immunoreactivity was detected using an enhanced chemiluminescence detection kit (ECL, Amersham).

### 4.8. Separation of Nuclear and Cytoplasmic Fractions

The cells were rinsed in PBS and then collected into Eppendorf tubes. A volume of 0.5 mL of Solution A (10 mM HEPES, 1.5 mM MgCl_2_, 10 mM KCl, 0.5 mM DTT, 0.05% NP40, pH 7.9) was then added, after which the cells were centrifuged at 805× *g* for 10 min at 4 °C. The supernatant containing mostly cytoplasmic constituents was then removed and transferred to another tube. To yield a nuclear pellet, 0.4 mL of solution B (5 mM HEPES, 1.5 mM MgCl_2_, 0.2 mM EDTA, 0.5 mM DTT, 26% glycerol (*v*/*v*), 300 mM NaCl, pH 7.9) was added and the tubes were mixed thoroughly and placed on a small rotator shaker for 15 min. Finally, the mixture was centrifuged at 24,000× *g* for 20 min at 4 °C. The supernatant containing the proteins from the nuclear extract was removed and carefully transferred into a fresh tube. The nuclear and cytosol extracts were frozen at −80 °C in aliquots until the western blot analysis was performed. The protein content of each sample was determined using the BCA protein assay kit (Thermo Scientific, Waltham, MA, USA).

### 4.9. Confocal Microscopy Analysis

The BMMs were plated in 96-well plates and stimulated with 250 ng/mL of M-CSF and 250 ng/mL of RANKL for 3 days in the absence. The cells were then fixed with 4% paraformaldehyde (PFA) for 10 min, permeated with 0.1% Triton X-100 for 5 min, and stained with DAPI. The immunolabeled cells were counterstained with 4′,6′-diamidino-2-phenylindole (DAPI) provided in ProLong Gold antifade mounting medium (Invitrogen; Thermo Fisher Scientific, Inc., Waltham, MA, USA) to visualize nuclear morphology. Digital images were acquired at the Korea Basic Science Institute Gwangju Center using a TCS SP5 AOBS laser-scanning confocal microscope (Leica Microsystems, Heidelberg, Germany). Fluorescence images were obtained by sequential z-stage scanning in two channels (DAPI and Alexa Fluor-488); z-stacks were compiled into individual images.

### 4.10. Mice

Five-week-old female mice (BL-6; Orient Bio Co. LTD, Seoul, Korea) were housed under controlled light conditions and fed ad libitum. All experimental procedures involving animals were performed in compliance with institutional and governmental requirements and approved by the Institutional Animal Care and Use Committee (CIACUC2018-S0012-1) of Chosun University, Gwangju, Korea.

Fifteen mice were equally divided into three groups. The control group was intraperitoneally injected with PBS; The mRANKL-WT group was given mRANKL-WT (2 mg/kg) in PBS and the mRANKL-WT+mRANKL-MT group was given mRANKL-WT(2 mg/kg) with mRANKL-MT(2 mg/kg) in PBS at 24-hrs intervals for 2 days. Mice were sacrificed on the third day according to the indicated schedule (Figure 4A).

### 4.11. Micro-Computed Tomography (CT) Imaging and Data Acquisition

Micro-CT scanning for the distal femur was distally initiated at the level of the growth plate using a Quantum GX micro-CT imaging system (PerkinElmer, Hopkinton, MA, USA) located at the Korea Basic Science Institute in Gwangju, Korea. The scanning X-ray source was set to 90 kV and 88 mA with a field of view of 10 mm (voxel size, 20 μm; scanning time, 4 min). The 3D imaging was performed via 3D Viewer, an existing software within the Quantum GX system. The resolution was set at 4.5 μm and images were obtained. Following scanning, the structural parameters for the trabecular bone were analyzed using the Analyze 12.0 software (AnalyzeDirect, Overland Park, KS, USA). The mineral density of the femur was estimated using a hydroxyapatite (HA) phantom (QRM-MicroCT-HA, Quality Assurance in Radiology and Medicine GmbH, Möhrendorf, Germany), which was scanned using the same parameters. The bone mineral density (BMD), % of bone volume (bone volume/tissue volume, %), trabecular number (Tb. N.), trabecular separation (Tb. Sp.), and trabecular thickness of the femurs were calculated using the ROI tool. Parameters values are shown as mean ± standard deviation (SD).

### 4.12. Histological Analysis of Mouse Tissues

Mouse femur tissues were fixed in cold 4% PFA. The bone tissue was first decalcified using a 0.5 M EDTA solution before processing onto the histological slides. The decalcified bones were cut at the midpoint and embedded in paraffin blocks. The tissues were then stained with hematoxylin & eosin (H&E) or TRAP and images were acquired using an ECLIPSE Ts2R inverted microscope (Nikon, Tokyo, Japan).

### 4.13. Statistical Analysis

All in vitro studies were conducted in at least triplicate. All quantitative results are presented as mean ± SD. The statistical significance of the differences in primary cell comparisons and the data from all animal studies were analyzed with two-way repeated measure ANOVA with Bonferroni multiple-comparisons test. All reported *p* values were two-sided, and those <0.05 were considered statistically significant. All statistical analyses were performed using the GraphPad Prism Version 7 software (GraphPad Software Inc., San Diego, CA, USA).

## Data Availability

All requests for raw and analyzed data and materials will be promptly reviewed to verify whether the request is subject to any intellectual property or confidentiality obligations by the corresponding author and the Chosun University, Republic of Korea.

## Abbreviations

RANKL	Receptor activator of NF-kB ligand
M-CSF	Macrophage colony-stimulating factor
LGR4	Leucine Rich Repeat Containing G Protein-Coupled Receptor 4
TRAP	Tartrate-resistant acid phosphate
NFATc1	Nuclear factor of activated T cells c1
OSCAR	Osteoclast-associated receptor
DC-STAMP	Dedritic cell specific transmembrane protein
BMMs	Bone marrow-derived monocytes
OPG	Osteoprotogerin
BMD	Bone Mineral Density
BV/TV	Bone volume over total volume
Tb. N	Trabecular number
Tb. Th	Trabecular thickness
CT	Computerized tomography
VOI	Volume of interest
DAPI	4′,6-diamidino-2-phenylindole
